# Phosphorylation of the Leukemic Oncoprotein EVI1 on Serine 196 Modulates DNA Binding, Transcriptional Repression and Transforming Ability

**DOI:** 10.1371/journal.pone.0066510

**Published:** 2013-06-12

**Authors:** Daniel J. White, Richard D. Unwin, Eric Bindels, Andrew Pierce, Hsiang-Ying Teng, Joanne Muter, Brigit Greystoke, Tim D. Somerville, John Griffiths, Simon Lovell, Tim C. P. Somervaille, Ruud Delwel, Anthony D. Whetton, Stefan Meyer

**Affiliations:** 1 Stem Cell and Leukaemia Proteomics Laboratory, University of Manchester, Manchester Academic Health Science Centre, Manchester, United Kingdom; 2 Department of Hematology, Erasmus University, Rotterdam, The Netherlands; 3 Cancer Research UK Leukaemia Biology Laboratory, Paterson Institute of Cancer Research, Manchester, United Kingdom; 4 Faculty of Life Sciences, University of Manchester, Academic Health Science Centre, Manchester, United Kingdom; 5 Paediatric Haematology and Oncology, Royal Manchester Children’s Hospital and The Christie NHS Foundation Trust, Manchester Academic Health Science Centre, Manchester, United Kingdom; Université Paris-Diderot, France

## Abstract

The *EVI1* (ecotropic viral integration site 1) gene at 3q26 codes for a transcriptional regulator with an essential role in haematopoiesis. Overexpression of *EVI1* in acute myeloid leukaemia (AML) is frequently associated with 3q26 rearrangements and confers extremely poor prognosis. EVI1 mediates transcriptional regulation, signalling, and epigenetic modifications by interacting with DNA, proteins and protein complexes. To explore to what extent protein phosphorylation impacts on EVI1 functions, we analysed endogenous EVI1 protein from a high EVI1 expressing Fanconi anaemia (FA) derived AML cell line. Mass spectrometric analysis of immunoprecipitated EVI1 revealed phosphorylation at serine 196 (S196) in the sixth zinc finger of the N-terminal zinc finger domain. Mutated EVI1 with an aspartate substitution at serine 196 (S196D), which mimics serine phosphorylation of this site, exhibited reduced DNA-binding and transcriptional repression from a gene promotor selectively targeted by the N-terminal zinc finger domain. Forced expression of the S196D mutant significantly reduced EVI1 mediated transformation of Rat1 fibroblasts. While EVI1-mediated serial replating of murine haematopoietic progenitors was maintained by EVI1-S196D, this was associated with significantly higher *Evi1-*trancript levels compared with WT-EVI1 or EVI1-S196A, mimicking S196 non-phosphorylated EVI1. These data suggest that EVI1 function is modulated by phosphorylation of the first zinc finger domain.

## Introduction

The *Ecotropic viral integration site-1* gene (*EVI1*), at the MECOM (MDS-EVI1 complex) locus at 3q26.2 encodes a nuclear protein with an essential role in haematopoiesis [1]. Overexpression of *EVI1* in acute myeloid leukaemia (AML) is commonly a result of chromosomal rearrangements involving the 3q26.2 region and is associated with an extremely poor clinical outcome [2], [3]. *EVI1* overexpression is in particular linked to leukemic transformation in individuals with Fanconi Anaemia (FA), which is an inherited chromosomal fragility disorder with predisposition to AML [4], [5]. EVI1 functions as a transcriptional regulator with two zinc finger domains that recognize specific genomic DNA target sequences [6], [7] and mediates interaction of DNA with chromatin modifying proteins and protein complexes to regulate gene expression [8], [9], [10]. EVI1 regulated genes have been reported to include *Pbx1* and *Fos* [11], [12]. The oncogenic potential of overexpressed EVI1 is reflected by the transformation of Rat1 fibroblasts to anchorage independent growth [13], as well as EVI1 induced immortalization of primary bone marrow cells, and induction of myeloid dysplastic syndrome in mice [13], [14], [15]. EVI1-mediated transformation *in vitro* and *in vivo,* however, has been shown to be abrogated when interactions between EVI1 and its co-regulatory protein complexes, or its target DNA sequences are disrupted [16], [17]. Protein phosphorylation has been shown to be an important modulator of transcriptional regulators in development, haematopoiesis and differentiation [18], [19], [20]. Phosphorylation of EVI1 was first identified by metabolic labelling studies more than 20 years ago [21], and more recently in large scale proteomic studies [22], [23], [24]. To test the hypothesis that EVI1 phosphorylation plays a role in modulating EVI1 protein function, we analysed endogenously expressed EVI1 from the FA-derived AML cell line SB1690CB by mass spectrometry and report here on the functional analysis of EVI1 phosphorylation on serine 196 (S196).

## Results

### EVI1 is phosphorylated on Serine 196

**Figure 1:**
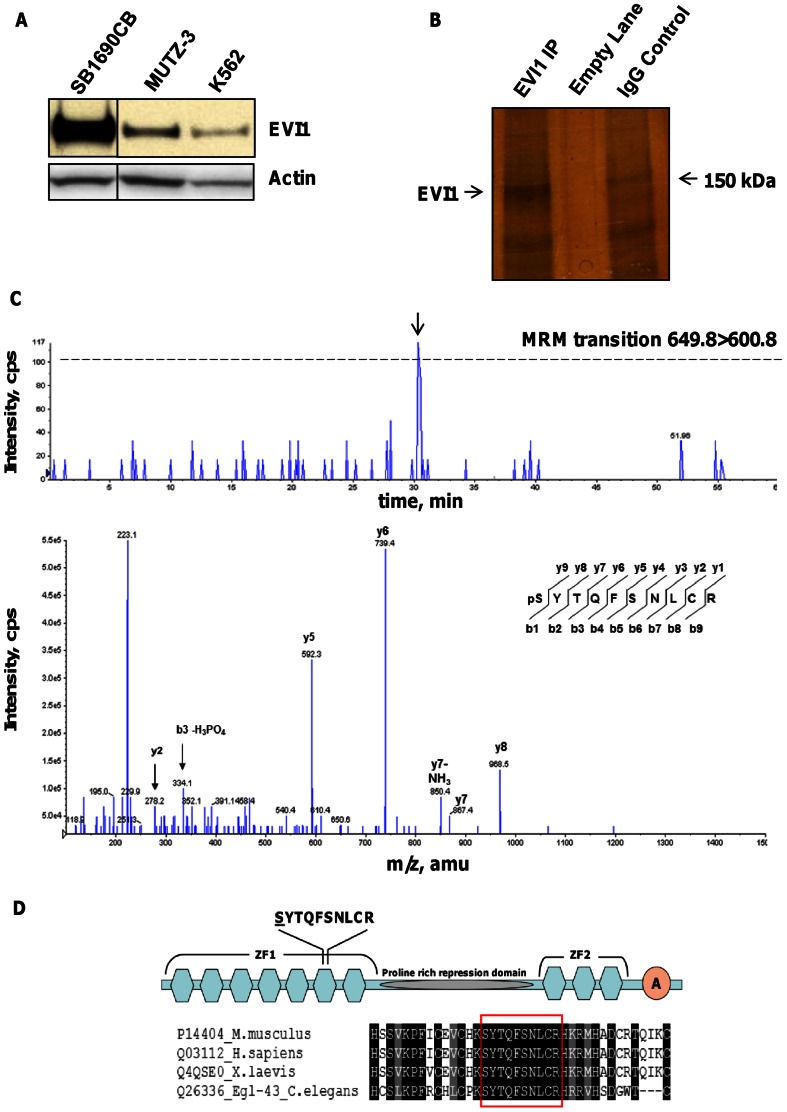
EVI1 is phosphorylated on Serine 196 in SB1690CB cells. (**A**) Western blot detection of EVI1 from whole cell lysate of leukemia cell lines SB1690CB, MUTZ-3 and K562. A vertical line has been inserted to indicate a repositioned gel lane. (**B**) Silver staining of immunoprecipitated EVI1 protein (arrow) from large scale SB1690CB cultures. Negative control lane showing immunoprecipitation with an irrelevant IgG antibody. (**C**) Extracted ion chromatograph showing signal intensity at time for the MRM transition 649.8>600.8 designed to selectively detect the EVI1 peptide SYTQFSNLCR with a single serine or threonine phosphorylation. The threshold for triggering fragmentation and sequencing of the peptide was 100 counts per second (cps) and is indicated with a dashed line. A peak exceeding this threshold was eluted at 30.49 min and triggered fragmentation of the peptide (arrow). Lower panel: product ion spectrum generated by peptide fragmentation. Detected y- or b-type fragment ions are indicated. Sequence of the EVI1 phosphopeptide with the b-ion and y-ion series and position of the phosphorylated serine residue at the N-terminus of the peptide is shown. (**D**) Illustration showing the location of phosphorylated peptide within the EVI1 protein, with hexagons for zinc finger (ZF) motifs and acidic region, A-circle, at the carboxy terminal region (modified from [48]). Underlined is the serine residue at site of phosphorylation. Alignment was carried out using the t-coffee algorithm at www.t-coffee.org [49]. Shown is alignment to human EVI1 amino acids H182 to C219 with the phosphorylated peptide boxed in red.

We detected high EVI1 protein expression in the FA-derived AML cell line SB1690CB, in which FA-associated 3q gains result in high *EVI1* transcript levels [5] (**Figure 1A**). This enabled immunoprecipitation of sufficient endogenous EVI1 protein to perform mass spectrometric analysis (**Figure 1B**). A multiple reaction monitoring-initiated detection and sequencing (MIDAS) scan for putative phosphopeptides detected a signal associated with potential phosphorylation of the peptide SYTQFSNLCR. Phosphorylation at serine 196 (S196) of EVI1 was confirmed by analysis of the MRM-triggered MS/MS spectrum from the putative phosphopeptide (**Figure 1C**). S196 is part of the evolutionarily conserved sixth zinc-finger motif within the N-terminal zinc-finger domain of EVI1 (**Figure 1D**), which specifically binds to the DNA sequence GA(T/C)AAGA(T/C)AAGATAA [6]. In addition, we confirmed previously described carboxy-terminal phosphorylation (S858 and S860) of EVI1 (data not shown) [22], [23].

### Impact of S196 phosphorylation on EVI1 protein structure

In order to determine the structural context of the phosphorylated serine 196, a comparative model of the sixth EVI1 zinc finger domain was generated using standard techniques with a model of the human C2H2 type zinc finger of protein 484 (pdb code 2EMH) as a template.

**Figure 2:**
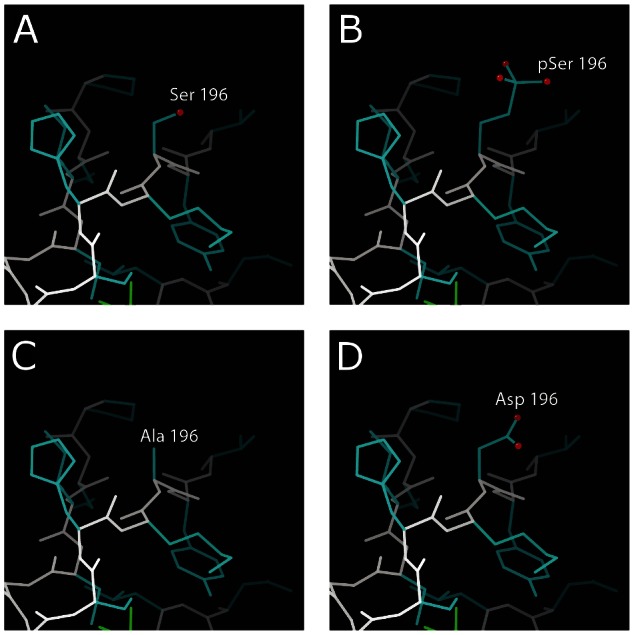
Structural context of phosphorylation and substitutions at EVI1 S196 . Main chain is shown in white and side chains in blue. Oxygen atoms on residue 196 are indicated by red spheres. (**A**) The zinc finger domain with unmodified serine, (**B**) phosphorylated serine, (**C**) aspartate substitution and (**D**) alanine substitution. All residues can adopt a similar conformation and extend into the solvent.

Serine 196 is on the surface of the zinc finger domain, which allows it to accommodate the phosphate group (**Figure 2A and B**). The impact of alanine and aspartate substitution at S196, used for functional analysis of S196 phosphorylation, was also modelled (**Figure 2C and D**). Given the solvent-exposed nature of this site, both aspartate and alanine can also be accommodated with no van der Waals overlaps. This suggests that the structural integrity of the proximal EVI1 zinc finger domain is maintained in the S196 phosphorylated, and alanine or aspartate substituted protein.

### Phosphorylation of S196 modulates DNA binding and transcriptional repression by EVI1

**Figure 3:**
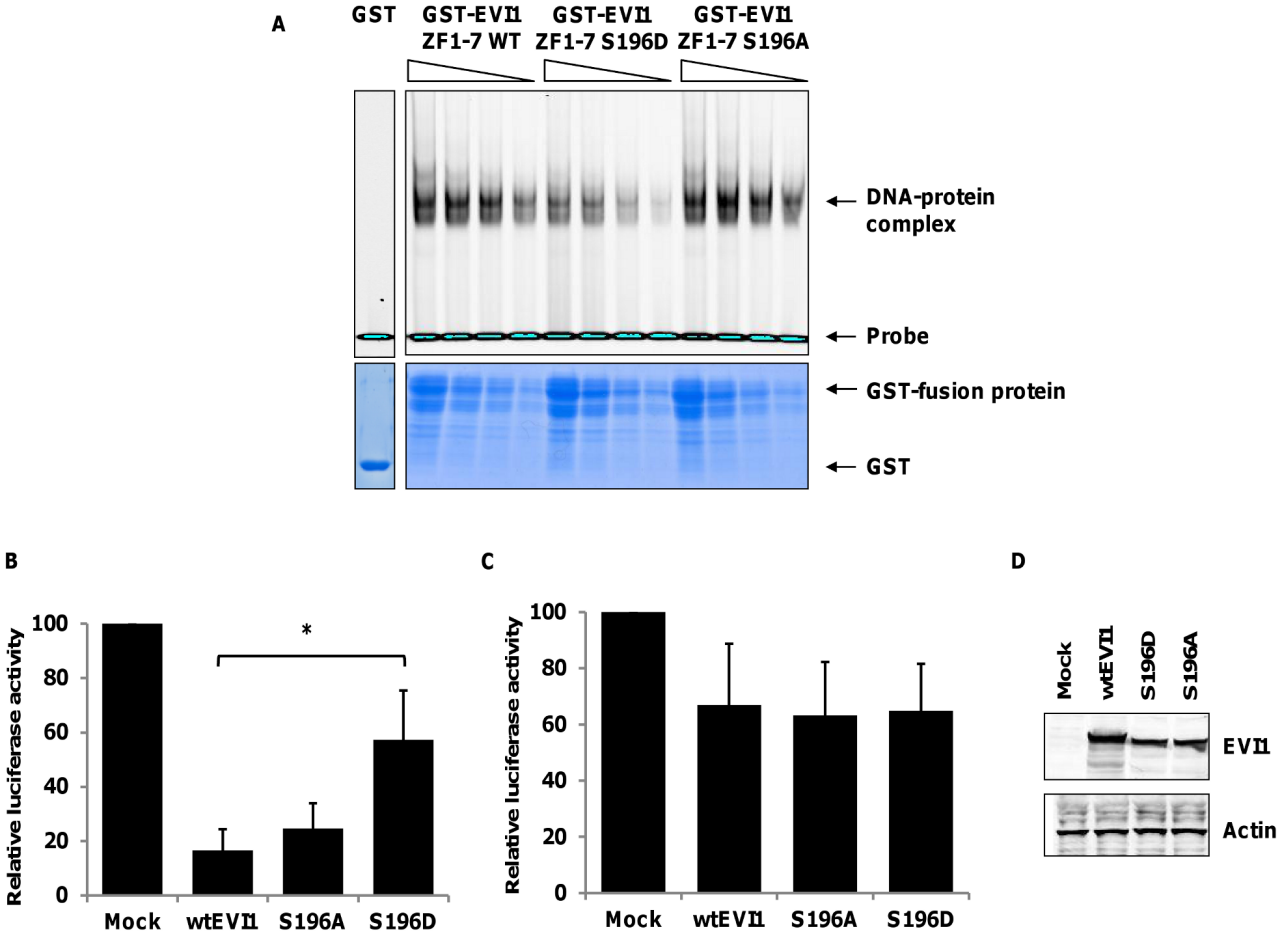
EVI1 S196 phosphorylation disrupts DNA binding and transcriptional repression. (**A**) Electro-mobility shift assay (EMSA) with GST alone or GST-Evi1 (ZF1-7) fusion proteins corresponding to WT or mutant Evi1 mimicking non-phosphorylated (S196A) or phosphorylated serine 196 (S196D) (upper panel). The DNA-protein complex and the free, labelled DNA probe are indicated. Lower panel: GST-fusion proteins separated on a 12.5% (w/v) SDS-PAGE gel and stained with colloidal coomassie blue. Specificity of protein/DNA interaction was confirmed by incubation with excess concentration of unlabelled random DNA substrate (data not shown). Graphs show mean (±s.d.,n = 4) luciferase activity relative to an empty vector control from reporter gene assays using either **(B)** a PLZF-luc reporter or **(C)** a *Fos* reporter co-transfected into HEK 293 cells with wild-type Evi1, S196 mutants or empty vector control. Luciferase activities were normalised against Renilla activity and calibrated to the empty vector control, *P<0.01, ns = non-significant (**D**) Western blotting of wild-type and mutant Evi1 protein levels in the reporter gene assays.

To investigate whether modulation of EVI1 S196 modifies the interaction with its target DNA sequences, we generated GST-fusion proteins with GST fused to the first zinc-finger domain of murine EVI1 [6], in which S196 was substituted with either aspartate (S196D) to mimic constitutively phosphorylated serine, or alanine (S196A), which mimics non-phosphorylated serine. We confirmed the strong interaction between the wtEVI1-GST fusion protein and the double stranded oligonucleotide containing the target DNA sequence GA(T/C)AAGA(T/C)AAGATAA recognized by the first zinc-finger domain of EVI1 by EMSA (**Figure 3A**) [6]. The S196A-substituted mutant EVI1-fusion protein mimicking non-phosphorylated EVI1 had equivalent binding to the target sequence as wtEVI1. However, the phosphomimetic EVI1-fusion protein showed decreased DNA binding (**Figure3A**), suggesting that phosphorylation status of S196 modulates DNA binding of EVI1. We next assessed the impact of full length EVI1-S196 mutants on transcriptional repression using luciferase reporter constructs with *PLZF* and *Fos* promoters. *In vitro*, *PLZF* is regulated by the proximal Evi1 zinc finger domain [25], while the *Fos* promoter is regulated by the distal zinc-finger domain of EVI1 [11]. Wt-EVI1 repressed transcription from both reporter constructs (**Figure 3** **B and C**). The Evi1-S196A mutant mimicking non-phosphorylated Evi1 repressed transcription from both reporter gene constructs as efficiently as wt-EVI1. In contrast, the phosphomimetic Evi1-S196D mutant repressed transcription from the *Fos* promoter, but repression from the *PLZF* promotor was significantly abrogated, implying that S196 phosphorylation impacts selectively on transcriptional regulation by the first zinc finger domain of EVI1 (**Figure 3** **B and C**). Western blot analysis confirmed similar protein levels of both mutant and wt-EVI1 in the above described reporter assays (**Figure 3D**).

### Phosphorylation of S196 partially abrogates transforming ability of Evi1

**Figure 4:**
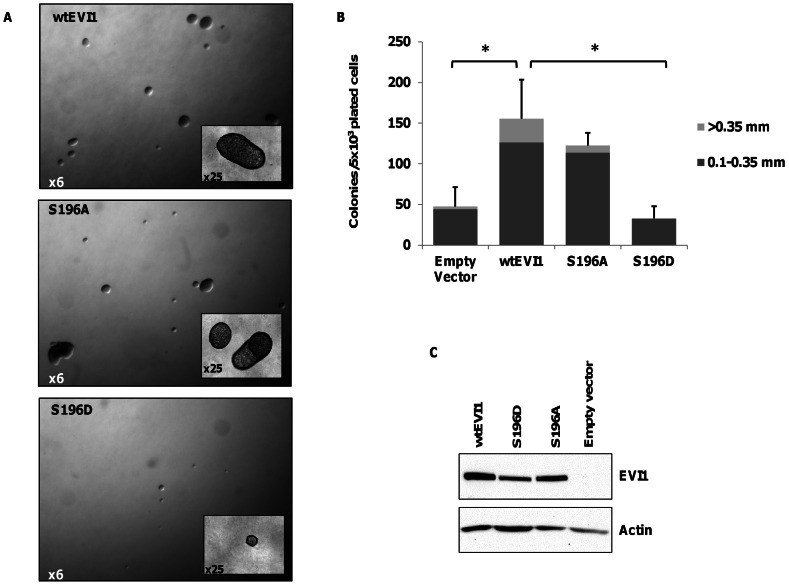
EVI1 S196 phosphorylation abrogates Rat1 transformation . (**A**) Representative photomicrographs of Rat1 fibroblast colonies transduced with wtEVI1 or S196 mutants (S196A and S196D) retroviral expression vectors. (**B**) Assays were scored after 24 days and colonies enumerated for number and size (mean±s.d., n = 3, *P< 0.05). **(C**) Detection of Evi1 protein in transduced Rat1 fibroblasts shown by western blot on whole cell lysates using the Evi1-SP137 antibody.

Transduction with EVI1 transforms Rat1 fibroblasts and enables anchorage independent growth in soft agar [13], [26]. Rat1 fibroblasts transduced with and stably expressing S196A mutant EVI1 mimicking non-phosphorylated Evi1 had similar colony forming capacity in soft agar as Rat1 fibroblasts transduced with wt-Evi1. However, Rat1 fibroblasts stably expressing the phosphomimetic EVI1 S196D mutation generated fewer (p<0.05, t-test) and smaller colonies compared to wt-EVI1(**Figure 4A** **+B**), despite similar Evi1 protein levels in all transduced Rat1 cells populations (**Figure 4C**). This implies that the ability of EVI1 to transform Rat1 fibroblasts is tightly regulated by the sixth zinc finger motif of the N-terminal zinc finger domain and is also modulated by S196 phosphorylation.

### Phosphorylation of S196 modulates EVI1 mediated replating capacity in murine haematopoietic progenitor cells

**Figure 5:**
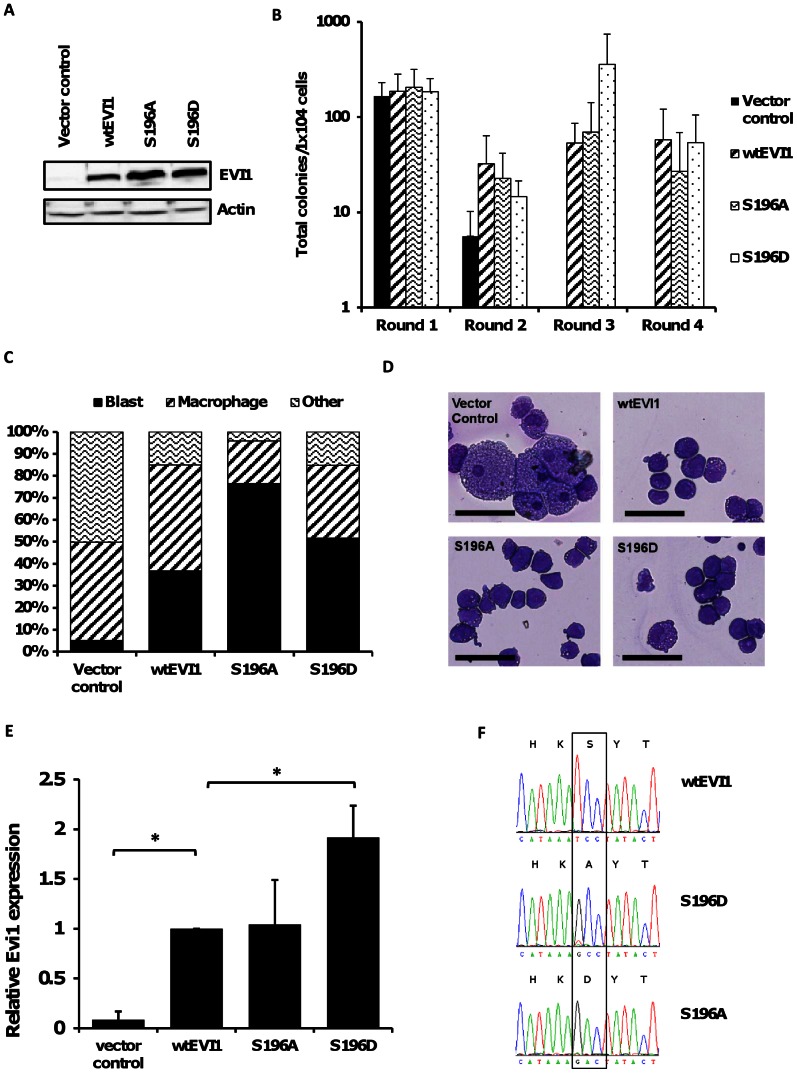
Serial replating of murine hematopoietic progenitor cells transduced with wtEvi1 and Evi1 phosphorylation site mutants. **(A)** Western blot analysis of Evi1 expression in PLAT-E cells two days after transfection with retroviral vectors encoding wtEVI1, Evi1-S196A, Evi1-S196D or empty vector control. **(B)** Total colony numbers (mean±s.d, n = 5) formed in serial replating assays by c-kit positive cells transduced with wtEVI1, S196A, S196D or control retroviral expression vectors. **(C)** Percentage of cell types (blast, macrophage or other) enumerated by morphological analysis of May-Grünwald Giemsa stained cytospins after the third round of replating. Mean, n = 4. Photomicrographs of May-Grünwald-Giemsa-stained cytospins after the third round of replating of transduced cells expressing WT or mutant EVI1 as shown. Scale bar indicates 50 µM. **(D)** Photomicrographs of May-Grünwald-Giemsa-stained cytospins after the third round of replating of transduced cells expressing WT or mutant EVI1 as shown. Scale bar indicates 50 µM. **(E)** Relative *Evi1* expression in c-kit+ cells after the first round of plating determined by qRT-PCR analysis. Mean±s.d, n = 4. *P<0.01. **(G)** Sanger-Sequencing of *Evi1* transcripts from c-kit+ cells after the first round of plating. Electropherograms illustrating DNA-sequence around codon encoding S196. Nucleotide and amino acid sequence (single letter amino acid code) are shown.

To investigate EVI1 S196 phosphorylation in the context of haematopoiesis, wt-EVI1 and phosphorylation site EVI1-mutants were expressed in c-kit+ murine primary bone marrow progenitors (**Figure 5A**). In serial replating assays progenitor cells transduced with control vector only formed colonies for two rounds of replating only, whereas cells with forced expression of exogenous wt-EVI1 replated beyond four rounds as previously described [13] (**Figure 5B**). The forced expression of either the EVI1-S196A or EVI1-S196D mutants enhanced replating capacity to the same extent as wt-EVI1 (**Figure 5B**). Morphological analysis of May-Grünwald-Giemsa stained cells at the end of the third round of replating showed persistence of immature cell types in the wt-EVI1, EVI1-S196A and EVI1-S196D transduced populations, whereas vector control cells had almost completely differentiated (**Figures 5C and D**). The comparable replating efficiency of EVI1-S196D compared with wt-EVI1 and EVI1-S196A demonstrates that the phospho-mutant retains sufficient function to confer serial replating, despite the impaired function that was demonstrated in the Rat1 fibroblasts assay. Since EVI1-mediated serial replating of hematopoietic progenitors appears highly dependent on EVI1-transcript levels, and is most effective with intermediate levels of EVI1 expression [13], we hypothesized that for replating of EVI1-S196D transduced progenitors higher EVI1-S196D expression levels might compensate for the impaired function that we demonstrate using in vitro and Rat1 fibroblast assays. Cells harvested from colonies transduced with EVI1-S196D showed significantly higher Evi1-expression after the first round of plating compared with either wt-EVI1 or S196A (p<0.01) (**Figure 5E**) by qRT-PCR analysis of Evi1 transcript levels. Sequence analysis of Evi1 transcripts in the S196A and S196D transduced cells showed that the Evi1 transcripts were expressed exclusively from the vector and not from the endogenous Mecom locus (**Figure 5F**)

## Discussion

Overexpression of *EVI1* is one of the most adverse prognostic markers in AML [2], [3]. Insight into EVI1 function might therefore have therapeutic implications for *EVI1* overexpressing AML. We hypothesised that the multiple functions of EVI1 might be partially modulated by protein phosphorylation. Therefore, we analysed endogenous EVI1 from an FA-derived AML cell line, which expresses high EVI1 transcript and protein levels as a result of FA-characteristic 3q gains [4], [27], [28]. This confirmed previously reported EVI1 phosphorylation sites (S858 and S860, data not shown), and detected a novel phosphorylation site at S196 within the sixth zinc finger of the first zinc finger domain of EVI1. Using the kinase substrate prediction software NetPhosK (http://www.cbs.dtu.dk/services/NetPhosK/) and NetworKin (http://networkin.info) several kinases were identified, which may be active on the EVI1 S196 site. These included: PKC, GSK3, CaM-II, CDC2, RSK, CKI, DNAPK, p38MAPK, PKA, PKG, ATM, CKII or NEK. As this phosphorylation site was detected in association with a defect in the FA-DNA damage response pathway, further investigations will focus on DNAPK, ATM and p38MAPK for this event, as these kinases in particular have been implicated in FA and the DNA damage response [29], [30], [31].

An *in silico* analysis of the structural impact of EVI1 S196 phosphorylation does not predict structural changes of this phosphorylation event on EVI1 structure. However, by functional analysis of the phosphomimetic mutant we demonstrated decreased DNA binding and selectively abolished transcriptional repression from a promotor targeted by the first EVI1 zinc finger, as the mutant *Evi1-S196D* had no effect on transcriptional repression of the *Fos* promotor, which is regulated by the second zinc finger domain [11]. In the Rat1 fibroblast assay a mutation mimicking phosphorylated Evi1-S196 nearly entirely abrogated the transforming ability of Evi1. By EVI1-mediated serial replating of murine haematopoietic progenitor cells we show that the phosphomimetic S196D mutant retained EVI1 function, and conferred serial replating as we also demonstrated for wt-EVI1 and EVI1-S196A. However, replating with Evi1-S196D was associated with higher transgene expression compared to wt-EVI1 or EVI1-S196A. Since only a narrow range of *EVI1* expression appears compatible with transformation and replating [12], [32] the significantly higher levels of S196D expression is consistent with impaired efficacy of the EVI1-S196D that we demonstrate *in vitro* and in Rat1 fibroblasts, which is compensated for by higher transcript levels.

In line with the observation that EVI1-mutations disrupting the first zinc-finger domain abolish the ability of EVI1 to induce myelodysplastic syndrome in mice [16], these data confirm a central role for the first-zinc finger domain of EVI1 in transformation, which is modulated by phosphorylation. EVI1-S196 phosphorylation appears to modulate EVI1 function by reducing affinity of the N-terminal zinc finger domain to specific DNA target sequences, without evidence of significantly affecting functions of other domains with respect to transcriptional regulation.

Zinc finger domain phosphorylation has been detected on other transcriptional regulators [33], [34], and phosphorylation mediated abrogation of DNA binding with effect on transforming ability has also been described for other leukemia associated transcription factors, including HOXA9**,** which intriguingly cooperates with EVI1 in leukaemogenesis [18], [35]. Given that EVI1 phosphorylation at S196 was detected in cells derived from AML originally arising in an FA-patient, our findings might be in particular relevant in the context of leukaemia associated with FA. However, methylation patterns associated with EVI1 overexpression overlap largely between this FA-derived AML cell line and sporadic AML [8]. It has previously been reported that DNA binding and transcriptional regulation of EVI1 is also regulated by acetylation via the P/CAF acetyltransferase, which prevents EVI1 binding to the promoter region of its target genes and modulates transcriptional activation [9], [36]. This implies complex regulation of EVI1 function by a spectrum of post-translational modifications. We have addressed the functional consequences of S196 phosphorylation in biochemical assays, the Rat1 model and murine haematopoietic progenitor cells, which suggests a modulatory effect of S196 phosphorylation, which is likely to be further confounded by tissue specificity and cellular differentiation. Therapeutically, it will be important to understand how these post-translational modifications functionally interact and to identify mediators and dynamics of S196 phosphorylation. Investigations are underway to identify the cell signalling pathways controlling these processes.

## Materials and Methods

### Cell lines

The EVI1 overexpressing FA derived AML cell line SB1690CB was established from the leukaemia of an FA patient [5], [27] and maintained under culture conditions as described previously [5], [8], [27]. Other cell lines were obtained from DSMZ (Deutsche Sammlung fr Mikroorganismen und Zellkulturen, Braunschweig, Germany). The leukemia cell lines MUTZ-3 and K562 were maintained as described previously [5], [27]. Rat1 cells were cultured as described before [13], [26], [37], [38]. GP-E86 cells lines were cultured in DMEM with 5% newborn calf serum, L-glutamine and penicillin/streptomycin. Plat-E and HEK-293T cells were cultured as previously described [39].

### Western Blot and Immunoprecipitation

EVI1 western blot and immunoprecipitation was carried out using standard protocols. An EVI1 antibody, raised against the N-terminal EVI1 epitope MKSEDYPHETMAPDI (Eurogentec, Liege, Belgium), and anti-EVI1 #2265 (Cell Signalling Technology, Boston, MA, USA) were used. For mass spectrometric analysis EVI1 was immunoprecipitated from protein lysates of large scale cultures of approximately 6×10^8^ SB1690CB cells. Evi1 detection in transformed Rat1 fibroblast was carried out with the anti-mouse Evi1-SP317 antibody (kind gift of Professor A. Perkins, Rochester, USA) [40].

### Mass spectrometry

EVI1 was analyzed using multiple reaction monitoring-initiated detection and sequencing (MIDAS) [41], [42]. MRM transitions were designed to detect EVI1 peptides (7 – 24 amino acids in length) with a single serine, threonine or tyrosine phosphorylation within a Q1 m/z range from 400 to 1300 and in both a double and triple charge state. A Q3 mass of either 216.0 Da or Q1 minus 98 Da was used to identify tyrosine or serine/threonine phosphorylation, respectively. Following gel separation of immunoprecipitated EVI1, the EVI1 containing band was excised, and digested with trypsin. Peptides were separated by liquid chromatography prior to MIDAS using electrospray mass spectrometry on a 4000 Q-TRAP mass spectrometer (AB Sciex, Toronto, Canada).

### Molecular modelling of the proximal EVI1 zinc finger domain

The phosphorylated serine is found in the first EVI1 zinc finger domain. No crystal structure is available for this domain. In order to assess the impact of phosphorylation or substitution of S196, a comparative model was generated using the human C2H2 type zinc finger of protein 484 (Database http://www.genome.jp/dbget-bin/www_bget?pdb:2EMH; pdb code 2EMH) as a template. For this, the sequences of EVI1 and the template structure were aligned using ClustalW [43] and 25 models built using Modeller [44]. The model with lowest discrete optimized protein energy (DOPE) score was selected. Models mimicking the specific mutations were built using KiNG (http://kinemage.biochem.duke.edu.) [45]. In each case all low-energy rotamers [46] were considered and the rotamer with fewest van der Waals overlap used. For the phosphorylated serine standard bond lengths were used.

### Plasmids

The retroviral Evi1 expression vector p50FL-neo and the bacterial expression vector pGEX-Evi1-ZF1-7 encoding the first seven zinc-fingers of Evi1 fused to GST have been described previously [6], [37], [38]. Mammalian expression constructs were generated by cloning Evi1 excised from p50FL-neo (Wild-type, S196A, and S196D) into a pCMV backbone. Substitution of S196 in pGEX-EVI1-ZF1-7, p50FL-neo and pMIG-FLAG-Evi1 to alanine (A), or aspartic acid (D) was achieved by site directed mutagenesis using the QuikChange® II XL Kit (Agilent, Santa Clara, USA). The mutagenesis primer oligonucleotides used were as follows: S196A FW GCCCTTTATCTGTGAGGTCTG CCATAAAGCCTATACTCAG; S196A RV CTGAGTATAGGCTTTATGGCAGACCTCACAGATAAAGGGC; and S196D FW GCCCTTTATCTGTGAGGTCTGCCATAAAGACTATACTCAG; S196D RV CTGAGTATAGTCTTTATGGCAGACCTCACAGATAAAGGGC. Mutations were confirmed by DNA sequencing. To generate pMIG-FLAG-Evi1 vectors a SalI FLAG-Evi1 fragment was excised from pCMV-FLAG-Evi1 (WT, S196A and S196D) and inserted into the XhoI site of pMIG. The reporter plasmids PLZF-luciferase, Fos-luciferase (a kind gift from Prof. Andrew Sharrocks, Manchester) and TK-Renilla luciferase have been previously described [25].

### Electromobilty shift assay (EMSA)

EMSA was carried out as described previously [6]. In brief, *E.coli* BL21 competent cells were transformed with pGEX-Evi1-ZF1-7 plasmids encoding for WT or S196-mutated GST-Evi1 fusion proteins. Fusion proteins were affinity purified using glutathione sepharose 4B and incubated with a double stranded DNA, sequence 5′ACCCATGGAGCTTTGAGCCCTGTTATCTTGTCAGGGAAGTGTACCATGGA’3, labelled at the 5′ end with IRDye. The reaction product was separated on a native 5% acrylamide gel and scanned on a Li-Cor Odyssey system (Li-Cor Biosciences, Lincoln NE, USA) to identify protein-DNA complexes.

### Reporter gene assay

Reporter gene assays were carried out in HEK-293T cells. Cells were plated out in 6-well plates at 3.5×10^5^ cells per well and co-transfected with 1 µg luciferase reporter plasmid, 1 µg effector plasmids (WT or mutants) and 5 ng TK-renilla luciferase using lipofectamine 2000 (Life Technologies, Carlsbad, CA,USA). After 48 hours cells were lysed and firefly and Renilla activity were assayed using the Dual-luciferase reporter assay (Promega, Madison, WI, USA) measured on a Victor^3^ plate reader (Perkin Elmer, Waltham,USA) or Flash’n Glow luminometer (Berthold Technologies, Bad Wildbad, Germany).

### Rat1 fibroblast transforming assay

Rat1 transduction with p50-M-X-neo, p50FL-neo, p50FL-196D-neo or p50FL-196A-neo retroviral constructs was carried out using the retroviral packaging cell line GP+E86, as described previously [13], [26], [37], [38]. In brief, Rat1 cells were seeded into 100-mm tissue culture plates at 1×10^6^ cells/plate and left to adhere. Medium was replaced with viral supernatant supplemented with polybrene (4 µg/mL) until Geneticin (G418, 400 µg/mL, Life Technologies) selection after 48 hours and maintained for 10 days to generate stable cell lines. Cells were seeded in soft agar and after 25 days quantified for colony number and size and documented using a standard inversion microscope.

### Serial replating of murine haematopoietic progenitors

This was carried out essentially as previously described [47]. Briefly, bone marrow was harvested from the femurs and tibias of 8-10 week old C57/BL6 mice. C-kit+ cell were isolated using an Automacs pro system (Miltenyi Biotechnology, Bergisch Gladbach, Germany), and pre-stimulated overnight in RPMI 1640 supplemented with recombinant murine IL-3 (10 ng/mL), GM-CSF (10 ng/mL), SCF (20 ng/mL) and IL-6 (10 ng/mL) in 20% fetal bovine serum, prior to transduction. Viral supernatant was generated by transfecting Plat-E packaging cells with *WT-Evi1* and *Evi1-S196* mutant pMIG-FLAG-Evi1 or control pMIG retroviral vectors using Fugene HD transfection reagent. C-kit+ cells were infected by spinoculation (30 minutes at 1250 x *g* and 32°C) with 8 µg/mL polybrene. Cells were cultured for an additional two days in pre-stimulation medium prior to FACS selection of GFP+ cells. Mean transduction efficiency for *WT-Evi1* was, 11.9% GFP+ cells; *Evi1-196A*, 13.8%, *Evi1-196D*, 14.0%; and pMIG control, 35.1%.

For replating 1×10^4^ GFP+ cells were plated out in Methocult M3231 medium (StemCell Technologies, Grenoble, France) supplemented with recombinant murine IL-3 (10 ng/mL), GM-CSF (10 ng/mL), SCF (20 ng/mL) and IL-6 (10 ng/mL). After 7 days colony number and type were enumerated. 1×10^4^ cells were then replated and colonies scored for at least three subsequent rounds of replating. Cytospin preparations and May-Grünwald Giemsa stainings were carried out using standard methods.

### EVI1 – transcript analysis

RNA was extracted from c-kit+ selected cells with an RNeasy Kit (Qiagen, Valencia, CA, USA). cDNA was generated using Omniscript reverse transcriptase (Qiagen) with random nonamers (Sigma, Dorset, UK) and quantitative RT-PCR reactions were carried out on an Applied Biosystems 7900 (Life Technologies). Evi1 qRT-PCR reactions consisted of Taqman universal mastermix (Life Technologies) and Taqman gene expression assay (Mm00514814_m1, Applied Biosystems). For sequence analysis an Evi1 PCR fragment was amplified from cDNA using the following oligonucleotides: FW, TGTGAAAACTGTGCCAAGGT; RV, ACTTAGATCCAGGGGCTGGT. Sanger sequencing of the purified PCR product was carried out using the standard techniques and the oligonucleotide primer AGGTAAGACCAGCAGGATGC.
